# Guatemala: How we create a welcoming workplace for our staff and patients

**Published:** 2018-07-31

**Authors:** Juan Francisco Yee

**Affiliations:** 1 Architect, MBA. Executive Director, Visualiza. Guatemala City, Guatemala.

Change and growth in our hospital can be stressful. We decided to address this by promoting staff motivation and team efficiency. Here is a short description of what we do at Visualiza in Guatemala.

We have created a hospital-wide engagement process to prevent and address problems of wide concern. The process is led by a service committee (SC), consisting of leaders from each of the departments, including counselling, outpatient, surgery, management. The SC is responsible for promoting improvement and motivation throughout the hospital.

## Interaction

The SC identifies problems that require improvement and carries out activities with all 135 staff members. Through quizzes, talks and games, they encourage groups to be creative and design solutions to the problems that have been identified. All are encouraged to make suggestions, and prizes are given to the most popular solutions in order to keep the process fun.

The SC also collaboratively develops – and then promotes – a profile setting out the qualities of a good employee. We train everyone in the “Magic of Service” and choose service tutors who observe and congratulate other staff members who offer high quality patient service.

## Continuity

After tutoring, the training is kept alive with reminders and activities to enforce the change. For example, we have lunchtime cinema every Tuesday to play a segment of a movie that brings out a situation that is thought-provoking and appreciated by the staff. We encourage everyone to participate.

The director encourages the SC to keep up with their motivational activities for the hospital by reviewing the SC agenda of activities, schedule and providing a budget.

## Outcomes

The willingness of staff members to express ideas has increased. Communication between managers and their team has improved. For the most part, team members are happy in their work area. This has a positive impact on teamwork and on the atmosphere the patients feel. We believe this positive culture-building attracts patients and boosts our overall quality of care.

Regional and institutional culture influences what kinds of motivation and team building activities are effective. How would you apply this example from Latin America to your own eye hospital or department? Please share your experience and ideas with us by writing to **editor@cehjournal.org**

**Figure:**
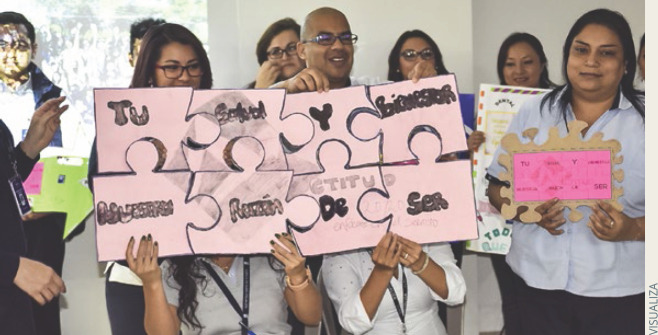
A group puzzle game spells out: ‘Your health and wellbeing is our reason for being’. GUATEMALA

